# Association Between EEG Activity and Motor Imagery Ability in Single-Joint Movements: A Pilot Study

**DOI:** 10.3390/brainsci16101008

**Published:** 2026-09-23

**Authors:** Takashi Inamoto, Masaya Ueda, Keita Ueno, Ryouhei Ishii, Yasuo Naito

**Affiliations:** 1 Graduate School of Comprehensive Rehabilitation, Osaka Prefecture University, Osaka 536-8525, Japan; precious.honored.i43.4@gmail.com (T.I.); rishii@omu.ac.jp (R.I.); 2 Faculty of Health Sciences, Kansai University of Health Sciences, Osaka 590-0482, Japan; 3 Graduate School of Rehabilitation Sciences, Osaka Metropolitan University, Osaka 536-8525, Japan; uedam@omu.ac.jp; 4 Department of Occupational Therapy, Morinomiya University of Medical Sciences, Osaka 559-8611, Japan; keita_ueno@morinomiya-u.ac.jp

**Keywords:** motor imagery, electroencephalography (EEG), kinesthetic motor imagery (KMI), visual motor imagery (VMI), motor imagery ability, single-joint movement, time–frequency analysis, cluster-based permutation test, pilot study

## Abstract

**Highlights:**

**What are the main findings?**

- In two hypothesis-driven analyses, kinesthetic KVIQ scores were associated with 6.5–13 Hz activity at O1 during KMI, and visual KVIQ scores were associated with post-task 10–16.5 Hz activity at F7 during VMI.
- The KMI–O1 pattern remained negative in leave-one-out and percentage-bend correlation analyses, whereas the VMI–F7 pattern was attenuated and was not significant in the percentage-bend correlation analysis.

**What are the implications of the main findings?**

- These exploratory pilot findings do not provide confirmatory evidence of distinct neural mechanisms underlying kinesthetic and visual motor imagery.
- Replication in a larger, independent study with prespecified analyses and objective measures of imagery performance is needed.

**Abstract:**

**Background/Objectives**: This exploratory pilot study examined whether motor imagery ability is associated with electroencephalography (EEG) oscillatory activity during single-joint motor imagery. **Methods**: Eleven healthy young adults performed motor execution, kinesthetic motor imagery (KMI), and visual motor imagery (VMI) of right wrist dorsiflexion. Motor imagery ability was assessed using the Kinesthetic and Visual Imagery Questionnaire (KVIQ). Time–frequency EEG data were analyzed using cluster-based permutation tests. Based on the study hypotheses, kinesthetic KVIQ scores were examined in relation to task-onset-locked KMI activity, and visual KVIQ scores were examined in relation to task-offset-locked VMI activity. A Bonferroni-adjusted threshold of *p* < 0.025 was applied across the two analyses. **Results**: No condition-level EEG differences were observed among motor execution, KMI, and VMI. Kinesthetic KVIQ scores were negatively associated with 6.5–13 Hz activity at O1 during KMI (*p* = 0.024), whereas visual KVIQ scores were positively associated with post-task 10–16.5 Hz activity at F7 during VMI (*p* = 0.023). Both met the Bonferroni-adjusted significance threshold. The corresponding descriptive Pearson correlations were r = −0.869 and r = 0.937. **Conclusions**: These exploratory findings should not be interpreted as confirmatory evidence of stable brain–behavior associations; the reported correlations are descriptive summaries based on clusters selected from the same dataset. Replication in a larger, independent study with prespecified analyses and objective measures of imagery performance is needed.

## 1. Introduction

Motor imagery is a cognitive process that involves the mental simulation of movement without actual bodily execution [1], and places substantial demands on working memory [2]. Motor imagery is typically classified into two modalities: kinesthetic motor imagery (KMI), which involves recalling somatosensory experiences associated with movement, and visual motor imagery (VMI), which involves mentally visualizing the movement. KMI emphasizes proprioceptive sensations such as muscle contraction and joint movement, whereas VMI can be experienced from a first-person or third-person perspective. Mental rehearsal using motor imagery has been shown to improve motor planning, coordination, and accuracy, thereby enhancing motor performance [3,4,5], and has been incorporated into neurorehabilitation programs across a range of neurological and musculoskeletal conditions because it requires no overt movement (see [6] for a comprehensive review). It is also increasingly utilized in brain–computer interface development.

In electroencephalographic (EEG) studies, two primary indices—event-related desynchronization (ERD) and event-related synchronization (ERS)—are used to assess task-related changes in local cortical activity. ERD generally reflects increased cortical excitability in task-related regions, whereas ERS indicates an inhibitory state of cortical neurons [7]. In particular, alpha-band ERS has been proposed to reflect active suppression of task-irrelevant visual cortical processing during selective attention [8]. Post-task increases in ERS are believed to represent neurophysiological recalibration toward baseline levels [9,10,11,12] and have also been associated with inhibition of task-relevant regions, sensory gating, and internal consolidation of information [13,14]. Large-scale studies have shown that ERS can persist for approximately 4–5 s following brief voluntary movements and about 3 s after postural control tasks [15,16], although the duration and magnitude of this post-task power increase vary with task complexity and duration [17,18]. In motor imagery research using EEG, the alpha (8–13 Hz) and beta (14–30 Hz) bands are commonly analyzed due to their known sensitivity to movement-related processes.

Although motor imagery evokes oscillatory changes similar to those observed during actual movement, the spatial distributions of these changes differ [19,20,21]. For example, Chholak et al. reported that KMI induces marked power increase in the alpha and beta bands over parietal regions, whereas VMI produces power increase predominantly over occipital areas [22]. In addition, beta-band synchronization over parietal sites is seen following both actual and imagined movements [7]. Converging evidence from other neuroimaging methods suggests that motor imagery recruits several brain regions that also participate in motor execution (ME), including the primary motor cortex, supplementary motor area, and parietal regions [23,24]. Additional areas such as the premotor cortex, posterior parietal cortex, and cerebellum are involved in the generation of imagery [25,26]. The prefrontal cortex, known for its roles in attentional control, motor inhibition, working memory, action prediction, and motor preparation, often shows greater activation during motor imagery than during ME [27,28,29,30,31,32]. This pattern has been systematically confirmed by a meta-analysis conducted by Hardwick et al. [24], which identified consistent activation of the left prefrontal cortex during motor imagery tasks. Moreover, certain novel or unfamiliar tasks have been shown to elicit even stronger engagement of the prefrontal cortex [33,34]. Consistent with these findings, KMI is preferentially associated with sensorimotor and cerebellar regions, whereas VMI recruits broader visual and frontoparietal networks [35,36,37,38,39,40]. Despite this substantial body of research, several important limitations remain. Many studies fail to clearly specify the motor imagery modality (KMI vs. VMI) and perspective (first-person vs. third-person), which impairs reproducibility and interpretability [41].

Moreover, individual differences in imagery ability—despite their known influence on neural responses—are frequently overlooked [42], even though substantial inter-individual variability, including persistent difficulty in generating vivid images, has long been recognized [43,44]. This variability partly reflects the fact that motor imagery requires constructing motor representations from prior movement experience and imposes substantial demands on attentional resources through the internal generation and manipulation of complex representations [6,45]. Consistent with this, effective-connectivity analyses show that supplementary motor area–to–prefrontal cortex information transfer occurs only during successful imagery trials, indicating that accurate imagery depends on preexisting, coherent motor representations [46]. Such individual differences also shape the neural strategies recruited during task performance: novices and individuals with lower KMI/VMI ability show greater occipital activation and altered occipital–frontal information flow than higher-ability imagers, consistent with compensatory recruitment of visually mediated strategies when internally generated representations are weak [47,48,49].

However, the relationship between individual differences in motor imagery ability and EEG activity during simple single-joint motor imagery tasks remains insufficiently characterized. This issue is relevant because single-joint movements are commonly used in motor imagery assessment and neurorehabilitation contexts, yet their neural correlates may differ from those observed during complex whole-body or multi-joint tasks. Therefore, as a proof-of-concept investigation, the present study was designed as a pilot study with a limited sample size to examine two theory-guided, exploratory associations between EEG oscillatory activity during a right wrist dorsiflexion imagery task and individual differences in kinesthetic and visual motor imagery ability. The findings were intended to generate hypotheses for subsequent preregistered replication rather than to provide confirmatory evidence of condition-specific neural mechanisms.

Based on previous findings, we specified two theory-guided, exploratory expectations. First, because single-joint imagery involves a more localized motor representation than whole-body imagery [50], we examined whether lower kinesthetic imagery ability would be associated with altered occipital alpha-band activity during task-onset-locked epochs, potentially reflecting differences in visual cortical involvement or suppression during task performance. Second, because VMI relies heavily on the working-memory- and attention-related processes supported by the prefrontal cortex described above, we examined whether individual differences in VMI ability would be reflected in post-task frontal alpha- and low-beta-band oscillatory activity during task-offset-locked epochs. This expectation was based on evidence that post-task oscillatory responses scale with working memory demand and may reflect neural disengagement following cognitive processing [9,10,11,12]. Accordingly, the sensor-level time–frequency EEG–behavior correlation analyses were restricted to two maps: (1) the association between kinesthetic KVIQ scores and occipital alpha-band activity in task-onset-locked epochs, and (2) the association between visual KVIQ scores and frontal post-task alpha- and low-beta-band activity in task-offset-locked epochs. No other sensor-level time–frequency EEG–behavior correlation maps involving different condition, epoch-type, or frequency-band combinations were analyzed. Given the small sample size, all findings were interpreted as preliminary and hypothesis-generating.

## 2. Materials and Methods

This study was designed as a preliminary cross-sectional pilot study with an experimental EEG component. The primary aim was to explore associations between motor imagery ability and EEG oscillatory activity during single-joint motor imagery, rather than to test confirmatory hypotheses.

### 2.1. Participant Recruitment and Eligibility

Thirteen healthy young adults, comprising undergraduate and graduate students, participated in this study. None reported a history of neurological or psychiatric disorders, as confirmed via a self-administered questionnaire before the experiment. Because this was a preliminary, proof-of-concept pilot study, no formal a priori sample size calculation for hypothesis testing was performed. The sample size was determined based on feasibility and data availability. Accordingly, the observed associations were interpreted as preliminary, hypothesis-generating findings rather than confirmatory evidence or reliable effect-size estimates for sample-size planning. Participants were excluded from EEG analyses if recordings showed bad channels, poor electrode contact, trigger failure, or insufficient data quality for time–frequency analysis. Data were collected between March 2022 and July 2023. Basic demographic information, including handedness, was collected. Handedness was determined using the Edinburgh Handedness Inventory (EHI) [51], which consists of ten items assessing hand preference in everyday activities. Responses were used to calculate a Laterality Quotient, classifying participants as right-handed, left-handed, or ambidextrous.

### 2.2. Fatigue Assessment

Subjective fatigue was assessed immediately before and after the experimental session using a Visual Analog Scale (VAS). Participants rated their current fatigue level on a 100 mm line anchored with “no fatigue” at the left end and “maximum imaginable fatigue” at the right end. Scores were measured in millimeters and converted to a 0–10 scale for analysis and presentation.

### 2.3. Motor Imagery Ability Assessment

Motor imagery ability was evaluated using the Kinesthetic and Visual Imagery Questionnaire (KVIQ), a widely used tool for assessing motor imagery ability [52]. In this study, we used the Japanese version of the KVIQ developed and validated by Nakano et al. [53]. KVIQ focuses on simple, single-joint movements of the upper and lower limbs to accommodate patients with severe motor impairments. Participants rated the vividness of imagined movements on a five-point scale for both kinesthetic and visual modalities. Visual imagery was conducted from a first-person perspective. The kinesthetic and visual scores each ranged from 5 to 25, and the total score ranged from 10 to 50. In the correlation analyses, the kinesthetic score was used for the KMI condition and the visual score was used for the VMI condition. To reduce participant burden, the 10-item short form (KVIQ-10), which focuses on single-joint movements of the upper and lower limbs, was administered.

### 2.4. Recordings

To minimize unintended visual influences on motor imagery performance, EEG data were recorded in a sound-attenuated, electrically shielded room under carefully controlled environmental conditions. Participants sat comfortably in a reclining chair, and the testing room was dimly lit with constant ambient lighting throughout all experimental conditions.

EEG signals were acquired using a Polymate AP-1000 biosignal recorder (Miyuki Giken Inc., Tokyo, Japan) with a 32-channel preamplifier and digitized on a laptop computer (CF-F9, Panasonic, Osaka, Japan). A 19-channel EEG cap (MCScap, East Medic Co., Ltd., Tokyo, Japan) fitted with sintered Ag/AgCl electrodes (MCScap E) was applied according to the international 10–20 system. Reference electrodes were attached to both earlobes (MCScap 1.5AE). The scalp was cleansed and conductive gel applied to maintain electrode impedance below 10 kΩ. EEG signals were sampled at 1000 Hz.

### 2.5. Experimental Procedure

Participants performed a right-hand wrist dorsiflexion task, which is not included in the KVIQ, to prevent prior familiarity with questionnaire items from influencing neural responses. The experimental task was conducted under three distinct conditions: Motor Execution (ME), Kinesthetic Motor Imagery (KMI), and Visual Motor Imagery (VMI). To ensure that all participants thoroughly understood the target movement before engaging in the imagery tasks, the ME condition was always performed first. The order of the KMI and VMI conditions was counterbalanced across participants. Verbal instructions matched those of the KVIQ:

- ME: “Please perform the movement.”
- KMI: “Please imagine the movement and focus on the strength of the associated sensations.”
- VMI: “Please imagine the movement from a first-person perspective and focus on the clarity of the image.”

In all conditions, participants were instructed to fixate on a central cross displayed on a 17-inch monitor positioned 60 cm from the eyes, with visual input limited to this static fixation cross presented on a uniform gray background and no additional visual stimuli or movement-related cues displayed during the task. Each condition comprised 20 trials, and each trial consisted of a 7 s task period followed by a 7 s rest period (Figure 1), resulting in a total duration of 280 s per condition.

The rest period was included to ensure clear separation between trials and to reduce potential carryover effects, during which participants were instructed to relax and prepare for the subsequent trial. Auditory tones delivered via speakers placed below the monitor signaled trial onset (1000 Hz, 500 ms) and offset (400 Hz, 100 ms) using the Stimulus Sequencer software (Miyuki Giken Inc., Tokyo, Japan), thereby clearly defining trial boundaries and ensuring that motor imagery was internally generated rather than visually guided.

Before data collection, all participants completed a practice session. They first viewed a demonstration video of the wrist dorsiflexion movement and then practiced 3 trials for each condition. This ensured that participants understood the task requirements and could accurately respond to auditory cues.

### 2.6. EEG Preprocessing

EEG data were first segmented by condition—ME, KMI, and VMI—and preprocessed using EEGLAB (v2023.0) in MATLAB R2022a. Continuous EEG recordings were band-pass-filtered between 1 and 40 Hz and notch-filtered at 60 Hz to remove line noise. Independent component analysis (ICA) was conducted using EEGLAB’s runica algorithm. Following the method described by Tonachini et al. [54], components classified as non-brain (e.g., muscle, eye, cardiac, line noise, channel noise, or other artifacts) with a probability ≥ 70% by ICLabel were removed. The remaining components were back-projected to reconstruct cleaned EEG signals. The EEG data were then re-referenced to the common average reference. Subsequently, epochs containing artifacts with amplitudes exceeding ±120 μV were automatically excluded. All epochs were further confirmed via visual inspection to ensure the absence of residual artifacts before analysis. Following these artifact rejection procedures, the mean number of trials retained for the time–frequency analysis was 18.6 ± 1.4 for the ME condition, 18.5 ± 1.2 for the KMI condition, and 18.2 ± 1.5 for the VMI condition (mean ± standard deviation).

### 2.7. Time–Frequency Analysis and Condition-Level Comparison

Cleaned data were imported into BESA Research 7.1 (BESA GmbH, Gräfelfing, Germany) for sensor-level time–frequency analysis. Two separate 10 s epochs were extracted from each trial: a task-onset-locked epoch (−5000 to 5000 ms relative to task onset) and a task-offset-locked epoch (−5000 to 5000 ms relative to task offset). In the task-onset-locked epoch, the interval from −5000 to 0 ms represented the pre-task resting period and the interval from 0 to 5000 ms represented the movement or imagery period. In the task-offset-locked epoch, the interval from −5000 to 0 ms represented the movement or imagery period, whereas the interval from 0 to 5000 ms represented the post-task resting period. Time–frequency analysis was performed separately for each condition and epoch alignment using complex demodulation, with a frequency resolution of 0.5 Hz and a temporal resolution of 100 ms. Analyses included data from 19 scalp electrodes over a time range of −5000 to 5000 ms and a frequency range of 1–40 Hz. At each frequency, time–frequency values were normalized to the mean amplitude during the corresponding pre-event interval and are presented as baseline-normalized relative amplitude changes (%). Specifically, at each time–frequency bin, the amplitude was expressed as the percentage change relative to the mean amplitude at the corresponding frequency during the pre-event interval. Accordingly, values from 0 to 5000 ms in the task-onset-locked analysis represent changes during movement or imagery relative to pre-task rest. In contrast, values from 0 to 5000 ms in the task-offset-locked analysis represent post-task changes relative to activity during movement or imagery.

Condition-level comparisons were conducted using BESA Statistics 2.0 (BESA GmbH, Gräfelfing, Germany), implementing a cluster-based, nonparametric permutation-testing procedure described by Bornfleth et al. [55]. A nonparametric cluster-based permutation one-way repeated-measures ANOVA was performed with one within-subject factor comprising three levels: motor execution (ME), kinesthetic motor imagery (KMI), and visual motor imagery (VMI). The analysis included 11 participants and no covariates. Time–frequency data from −5000 to 5000 ms and 1–40 Hz across 19 scalp electrodes were included in the analysis. During each permutation, condition labels were reassigned within participants to preserve the repeated-measures structure.

Clusters were formed from contiguous time–frequency–sensor points that exceeded a cluster-forming threshold of *p* < 0.05. Spatial adjacency was defined using a neighbor distance of 4 cm. No averaging was performed across time, frequency, or electrodes. For each observed cluster, the cluster statistic was calculated as the sum of the pointwise F-values within that cluster. Cluster-level significance was evaluated against a permutation-derived null distribution of cluster statistics obtained from 1000 permutations, using a significance threshold of *p* < 0.05. This procedure controlled for multiple comparisons across time, frequency, and sensor space within the ANOVA. Task-onset-locked and task-offset-locked epochs were analyzed separately; epoch alignment was not included as a factor, and no direct comparison between the two epoch alignments was performed.

### 2.8. Hypothesis-Driven Exploratory Correlation Analyses

Permutation-based correlation analyses were conducted in BESA Statistics 2.1 (BESA GmbH, Gräfelfing, Germany) to examine associations between EEG time–frequency activity and motor imagery ability. Based on the two theory-guided exploratory expectations described in the Introduction, only two sensor-level time–frequency EEG–behavior maps were analyzed: (1) the association between task-onset-locked KMI activity and kinesthetic KVIQ scores, and (2) the association between task-offset-locked VMI activity and visual KVIQ scores.

For each primary analysis, data from 19 scalp electrodes were examined from −5000 to 5000 ms and from 1 to 40 Hz. A neighbor distance of 4 cm, a cluster-forming threshold of *p* < 0.05, and 1000 permutations were used. During permutation testing, the correspondence between each participant’s time–frequency map and the corresponding KVIQ score was randomly reassigned to generate a null distribution of cluster-level statistics. Two-sided tests were used. The cluster-based permutation procedure controlled for multiple comparisons across time, frequency, and sensor space within each map, yielding cluster-level *p*-values. Because the complete analysis family comprised these two EEG–behavior maps only, a Bonferroni-adjusted significance threshold of 0.025 (0.05/2) was applied across the two maps.

### 2.9. Sensitivity Analyse Procedures

Sensitivity analyses were conducted for clusters identified in the two hypothesis-driven correlation analyses. For each cluster, a participant-specific mean EEG value was calculated across the time–frequency–sensor points comprising the cluster. Pearson correlations between the cluster-averaged EEG values and the corresponding KVIQ scores were calculated as descriptive summaries.

Leave-one-out sensitivity analyses were performed by excluding one participant at a time and recalculating the Pearson correlation. The clusters identified in the full sample were retained in all iterations; therefore, these analyses assessed the influence of individual observations and did not provide independent validation.

Percentage-bend correlations were calculated in R statistical software (version 4.3.3; R Foundation for Statistical Computing, Vienna, Austria) using the pbcor() function from the WRS2 package (version 1.1-7) with the default bending constant (β = 0.20). Ninety-five percent confidence intervals for the Pearson correlations were calculated using Fisher’s r-to-z transformation. Because the clusters and correlations were derived from the same small dataset, the resulting correlation coefficients and confidence intervals were treated as descriptive rather than as independent estimates of population-level associations.

## 3. Results

### 3.1. Participant Characteristics

Of the 13 initially recruited healthy young adults, two were excluded: one due to poor electrode contact and the other due to faulty task-trigger recordings. The final sample included 11 participants (6 males, 5 females; age range = 20–30 years; mean ± SD = 23.8 ± 3.4 years). Participant data are summarized in Table 1. Handedness, assessed via the Edinburgh Handedness Inventory, yielded a mean Laterality Quotient of 99.1 (SD = 3.0), indicating uniformly strong right-hand preference across participants.

### 3.2. Fatigue

Changes in subjective fatigue were evaluated using a paired-samples *t*-test. Subjective fatigue increased numerically from 5.42 ± 1.50 to 6.08 ± 2.12 on a 0–10 scale, but this change was not statistically significant (t(10) = 1.40, *p* = 0.191).

### 3.3. Motor Imagery Ability

Kinesthetic scores were 16.1 ± 4.7 (range: 8–22) and visual scores were 18.3 ± 3.6 (range: 11–25). These values fall within the range typically reported for healthy young adults in the KVIQ-10 validation studies, including the Japanese version, although the kinesthetic scores in the present sample were slightly lower on average than those reported by Nakano et al. [53].

### 3.4. Condition-Level EEG Comparison

The cluster-based permutation analysis of time–frequency EEG data revealed no statistically significant condition-level differences among ME, KMI, and VMI. The lowest cluster-level *p*-value was observed for a cluster over Fz from 0 to 2500 ms and 28 to 32.5 Hz (*p* = 0.052), which did not meet the significance criterion. Because the analysis did not yield a statistically significant cluster-level effect, no post hoc pairwise comparisons were performed. Additional channel-wise time–frequency representations for each condition are provided in the Supplementary Materials (Figures S1–S3) for qualitative reference.

### 3.5. Hypothesis-Driven Correlation Analyses

Two hypothesis-driven exploratory EEG–KVIQ correlation maps were analyzed. Kinesthetic KVIQ scores were examined in relation to task-onset-locked KMI activity, and visual KVIQ scores were examined in relation to task-offset-locked VMI activity. The cluster-based permutation procedure controlled multiple comparisons across time, frequency, and sensor space within each map. Because the complete analysis family comprised these two maps only, a Bonferroni-adjusted significance threshold of *p* < 0.025 was applied across the two analyses.

- In the task-onset-locked KMI analysis, a negative cluster-level association was identified at O1 from 1800 to 3400 ms and 6.5 to 13 Hz (Figure 2 and Figure 3; permutation-derived cluster-level *p* = 0.024). This result met the Bonferroni-adjusted significance threshold across the two analyses. The descriptive Pearson correlation between participant-level values averaged within the identified cluster and kinesthetic KVIQ scores was r = −0.869 (95% CI: −0.966 to −0.562; Figure 4).
- In the task-offset-locked VMI analysis, a positive cluster-level association was identified at F7 from 1000 to 3900 ms and 10 to 16.5 Hz, spanning the upper-alpha and low-beta frequency ranges (Figure 5 and Figure 6; permutation-derived cluster-level *p* = 0.023). This result met the Bonferroni-adjusted significance threshold across the two analyses. The descriptive Pearson correlation between participant-level values averaged within the identified cluster and visual KVIQ scores was r = 0.937 (95% CI: 0.770 to 0.984; Figure 7).

In the selected O1 cluster, higher kinesthetic KVIQ scores were associated with lower baseline-normalized relative amplitude changes during the task period. In the selected F7 cluster, higher visual KVIQ scores were associated with higher baseline-normalized relative amplitude changes during the post-task period relative to activity during the preceding imagery period. Because the clusters and correlations were derived from the same small dataset, the Pearson correlation coefficients and 95% confidence intervals should be interpreted as descriptive summaries rather than independent estimates of population-level associations.

### 3.6. Sensitivity Analyse Results

Leave-one-out sensitivity analyses were conducted using the O1 and F7 clusters identified in the full-sample hypothesis-driven analyses. For the KMI–O1 association, the Pearson correlation remained negative across all leave-one-out iterations (r = −0.804 to −0.916). The percentage-bend correlation was also negative (r_pb = −0.811, *p* = 0.002). For the VMI–F7 association, the Pearson correlation remained positive across all leave-one-out iterations (r = 0.883 to 0.958); however, the percentage-bend correlation was attenuated and was not statistically significant (r_pb = 0.489, *p* = 0.127). Because the clusters were fixed based on the full-sample analyses, these sensitivity analyses assessed the influence of individual observations and did not constitute independent validation.

## 4. Discussion

The present pilot study examined associations between individual motor imagery ability and EEG oscillatory activity during a single-joint task. The cluster-based permutation ANOVA detected no statistically significant differences in EEG oscillatory activity among the ME, KMI, and VMI conditions. This finding may reflect limited oscillatory differentiation during simple single-joint imagery or limited power due to the small sample size. In addition, because motor imagery is inherently unobservable and no trial-by-trial manipulation checks of imagery fidelity were obtained, the correlation analyses should be interpreted as reflecting individual variability within conditions rather than systematic differences between experimental conditions.

In the two hypothesis-driven analyses, higher kinesthetic imagery ability was associated with lower occipital alpha-range relative amplitude changes during KMI, whereas higher visual imagery ability was associated with greater post-task frontal alpha- and low-beta-range activity during VMI. Both cluster-level *p*-values met the Bonferroni-adjusted significance threshold across the two hypothesis-driven analyses. However, the VMI–F7 association was attenuated and was not statistically significant in the percentage-bend correlation analysis. Because the clusters and correlations were derived from the same small dataset, the cluster-averaged Pearson correlations and confidence intervals should be interpreted as descriptive summaries rather than independent estimates of population-level associations. The leave-one-out analyses assessed the influence of individual observations but did not provide independent validation. These findings require replication in a larger, independent sample.

### 4.1. KMI Ability and Occipital Oscillatory Modulation During Single-Joint Imagery

In the task-onset-locked KMI analysis, a negative cluster-level association was observed between kinesthetic KVIQ scores and 6.5–13 Hz activity at O1 during the imagery period (permutation-derived cluster-level *p* = 0.024). This result met the Bonferroni-adjusted significance threshold across the two hypothesis-driven analyses.

The O1 electrode site is presumed to overlie left occipital cortical regions [56,57,58]. Increases in occipital alpha amplitude during eyes-closed rest have often been discussed in relation to reduced visual cortical activity and suppression of visual information processing. Previous studies using whole-body movements have reported increased activity in occipital visual areas in individuals with lower KMI ability [47,48,50]. These studies have been interpreted as suggesting that such individuals may be less able to construct somatosensory-based motor representations during motor imagery and may therefore adopt compensatory visual strategies. One possible explanation for the divergent findings between the present and previous studies may be attributable to differences in task characteristics. Whole-body motor tasks used in earlier research require multisensory integration—including visual, vestibular, and somatosensory inputs [59]—and involve spatial structures and limb coordination associated with body movement, which include complex and diverse motion elements [60,61,62]. In such tasks, visual and spatial information may contribute more strongly to constructing or maintaining motor representations. In contrast, the single-joint repetitive movement task used in the present study involved a more localized target for body representation, potentially reducing the necessity and contribution of visual compensatory strategies compared with whole-body tasks.

The present findings provide a rationale for a preregistered replication examining whether kinesthetic imagery ability covaries with occipital alpha-range activity during single-joint imagery. However, oscillatory changes were quantified relative to a within-block pre-trial baseline, and scalp-level activity at O1 does not provide direct evidence of visual cortical suppression. Accordingly, this finding should not be interpreted as evidence of a specific cortical mechanism or compensatory visual strategy.

### 4.2. VMI-Related Association at the F7 Electrode Site

In the task-offset-locked VMI analysis, a positive cluster-level association was observed between visual KVIQ scores and 10–16.5 Hz activity at F7 during the post-task period (permutation-derived cluster-level *p* = 0.023). This result met the Bonferroni-adjusted significance threshold across the two hypothesis-driven analyses.

The F7 electrode site may reflect activity from underlying left frontal regions and is commonly associated with frontal and prefrontal regions [56,57,58]. The prefrontal cortex plays a central role in executive functions, including working memory, attentional control, distraction suppression, and the maintenance of internal representations [63,64,65], and has often been reported to be more active during motor imagery than during actual movement execution [24,30,31,32]. In other paradigms, increases in low-beta or alpha-band amplitude over prefrontal regions following task completion have been linked to processes such as cognitive disengagement, inhibitory control, interference filtering [66], and memory-related operations [67]. Post-task increases in frontal oscillatory power have been associated with disengagement of task-positive networks following cognitively demanding tasks, with response magnitude scaling with working memory load [9,10,11,16,17].

The VMI–F7 association was attenuated and was not statistically significant in the percentage-bend correlation analysis. In addition, the absence of condition-level effects in the cluster-based permutation ANOVA provided limited group-level evidence for condition-specific EEG differentiation. Therefore, this observation should be regarded as hypothesis-generating rather than as evidence for a stable VMI-specific or prefrontal mechanism. More broadly, the present findings should be interpreted as preliminary evidence that individual variability in motor imagery ability may be associated with differences in EEG oscillatory responses during motor imagery, not as evidence of condition-specific neural mechanisms.

### 4.3. Limitations

As a pilot study, these results should not be interpreted as providing definitive evidence of specific brain–behavior relationships but rather as preliminary observations requiring replication in adequately powered confirmatory studies.

Several limitations should be noted. First, the sample size was small (*n* = 11), which limited statistical power and increased uncertainty in effect size estimation. Accordingly, the reported 95% confidence intervals should be regarded as sample-dependent descriptions of uncertainty rather than precise estimates of population-level effects, as correlation estimates can be unstable in small samples. The observed correlation coefficients may therefore overestimate corresponding population-level effects or reflect sample-specific patterns. Both cluster-level *p*-values met the Bonferroni-adjusted significance threshold across the two hypothesis-driven analyses; nevertheless, the possibility of Type I errors cannot be completely excluded in this exploratory pilot study, and the reported associations should therefore be interpreted with appropriate caution. The VMI–F7 association was attenuated and was not statistically significant in the percentage-bend correlation analysis, suggesting sensitivity to influential observations. The KMI–O1 association remained negative in the sensitivity analyses; however, these analyses used clusters selected in the full dataset and did not provide independent validation. Accordingly, both patterns should be regarded as exploratory and hypothesis-generating. Second, motor imagery ability was assessed solely using the KVIQ, a self-report questionnaire, without complementary objective or behavioral measures such as mental chronometry. Because subjective ratings of imagery vividness may not fully reflect actual imagery performance, reliance on a single assessment method should be considered when interpreting the observed associations with EEG activity. In addition, no electromyographic or motion recordings were obtained, and no trial-level measures of imagery performance were collected. Therefore, subtle overt movements and adherence to the instructed imagery modality could not be objectively verified. Third, this study did not include an independent resting-state baseline. EEG amplitude changes were assessed relative to the pre-trial period within the experimental block; accordingly, the observed oscillatory modulations should not be interpreted as conventional event-related desynchronization or synchronization based on a stable resting baseline but rather as relative within-block amplitude modulations [68,69]. Fourth, the present analysis focused primarily on alpha- and beta-range activity and did not examine higher-frequency gamma-band activity, which may also contribute to cognitive processes [70,71,72,73]. Fifth, the participants were healthy young adults, and the task was limited to a simple single-joint movement. Therefore, the present findings may not generalize to older adults, individuals with neurological disorders, or more complex multi-joint movements relevant to activities of daily living. Future studies should include more diverse participant groups and ecologically valid motor imagery tasks [74].

Taken together, these limitations highlight the need for a preregistered confirmatory study with a larger, independent sample. Such a study should prespecify the primary outcome, frequency bands, electrode sites, and time window, and should complement KVIQ with objective measures of motor imagery performance, such as mental chronometry. Broader frequency ranges, including gamma-band activity, and network-level measures should also be considered.

## 5. Conclusions

This exploratory pilot study examined two hypothesis-driven associations between individual differences in motor imagery ability and EEG oscillatory modulation during single-joint motor imagery. Cluster-level associations were observed between kinesthetic KVIQ scores and activity at O1 during KMI and between visual KVIQ scores and post-task activity at F7 during VMI; both met the Bonferroni-adjusted significance threshold across the two hypothesis-driven analyses. The KMI–O1 pattern remained negative in leave-one-out sensitivity analyses and in the percentage-bend correlation analysis, whereas the VMI–F7 pattern was attenuated and was not statistically significant in the percentage-bend correlation analysis.

Given the small sample size (*n* = 11), the absence of condition-level EEG differences, and the fact that clusters were selected and summarized using the same dataset, these findings should be regarded as exploratory and hypothesis-generating rather than as confirmatory evidence of stable brain–behavior associations. The observed correlation coefficients and 95% confidence intervals should therefore be interpreted cautiously rather than as independent estimates of population-level effects. Replication in a larger, independent, adequately powered study with prespecified analytical windows and objective measures of imagery performance is needed before these associations can be considered confirmatory.

## Figures and Tables

**Figure 1:**
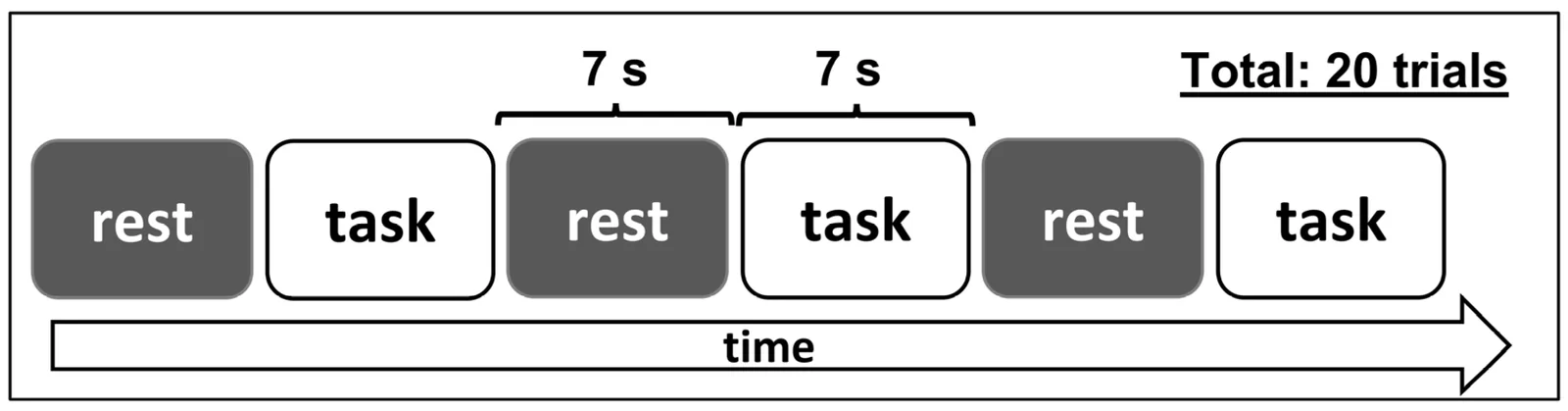
Schematic representation of the task order used in the experiment. Each condition included 20 trials with 7 s task and rest periods.

**Figure 2:**
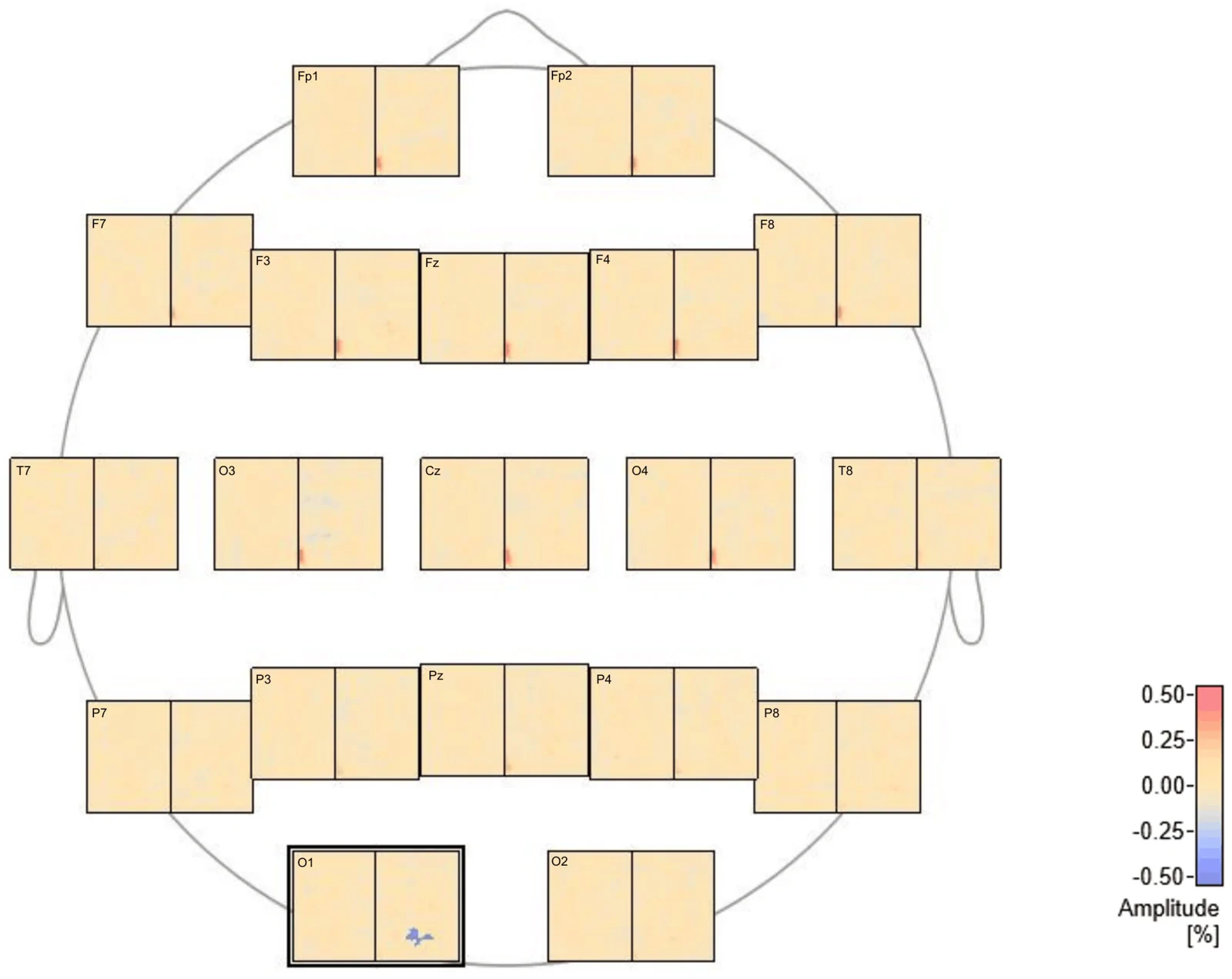
Sensor-level time–frequency results of the permutation-based correlation analysis between kinesthetic KVIQ scores and task-onset-locked KMI activity. Time–frequency maps for all 19 electrodes are shown, with each panel representing one electrode. A negative (blue) correlation cluster was observed at electrode O1.

**Figure 3:**
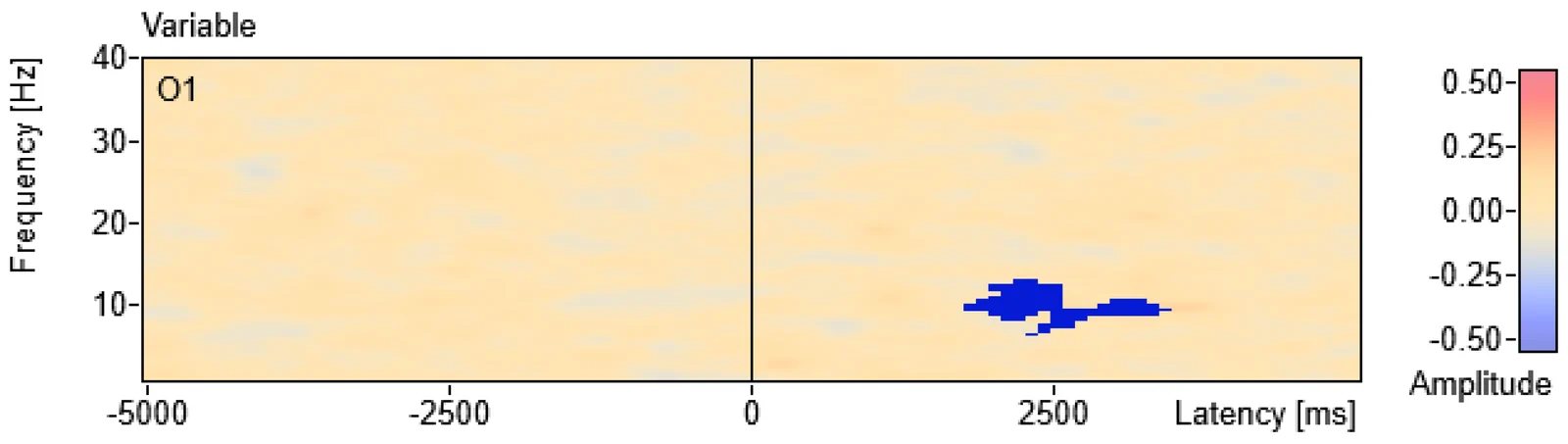
Enlarged time–frequency representation of the negative EEG–KVIQ correlation cluster identified at O1 in the task-onset-locked KMI analysis. The cluster extended from 1800 to 3400 ms and from 6.5 to 13 Hz (permutation-derived cluster-level *p* = 0.024). Time is shown on the horizontal axis and frequency on the vertical axis; 0 ms indicates task onset. The blue cluster indicates a negative correlation between kinesthetic KVIQ scores and baseline-normalized relative amplitude changes (%).

**Figure 4:**
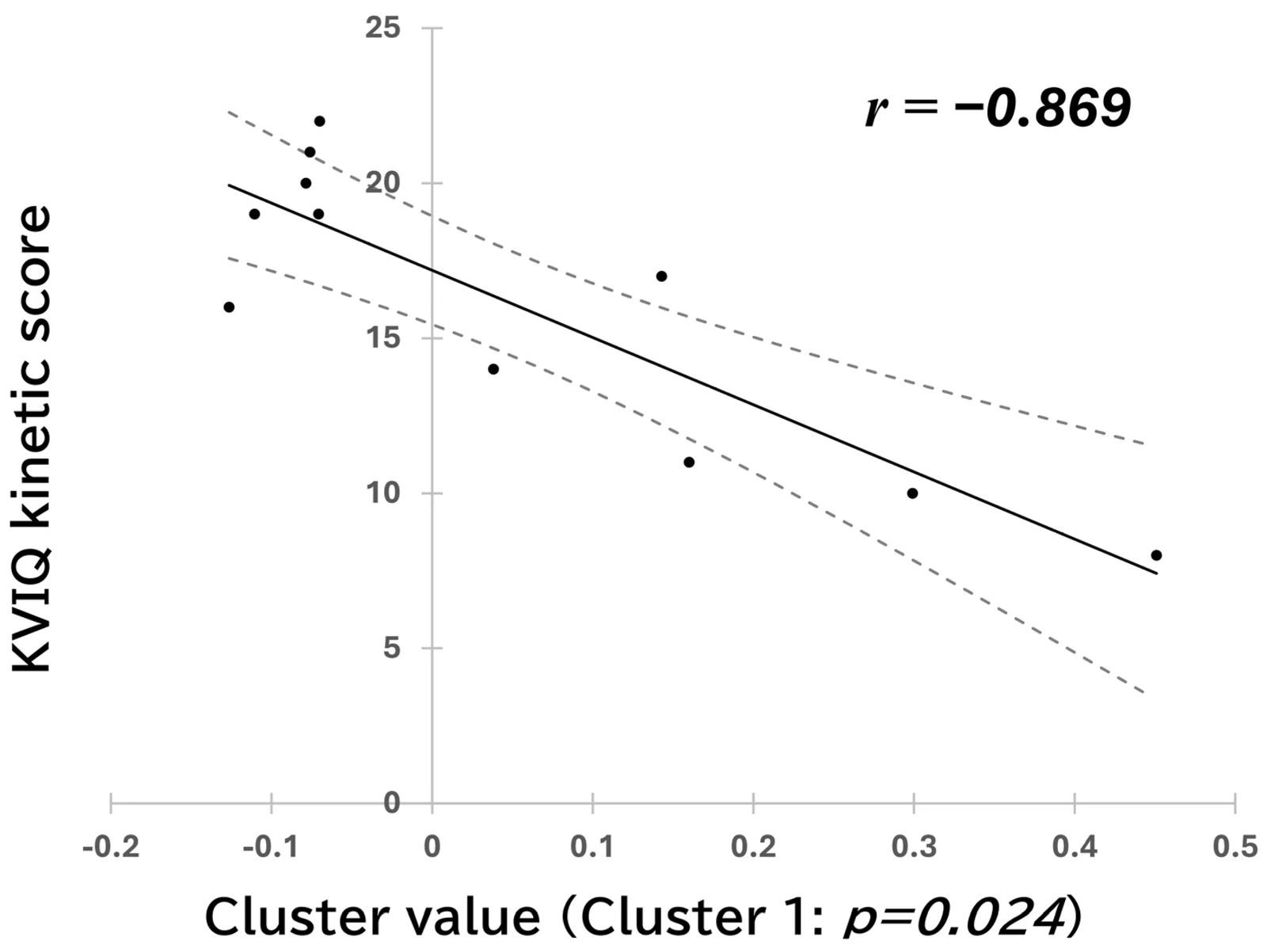
Scatter plot of kinesthetic KVIQ scores and mean baseline-normalized relative amplitude change (%) within the selected O1 time–frequency cluster in the task-onset-locked KMI analysis (1800–3400 ms; 6.5–13 Hz). The descriptive Pearson correlation was r = −0.869 (95% CI: −0.966 to −0.562); the permutation-derived cluster-level *p*-value was 0.024. The solid line indicates the least-squares regression line, and dashed lines indicate 95% confidence limits.

**Figure 5:**
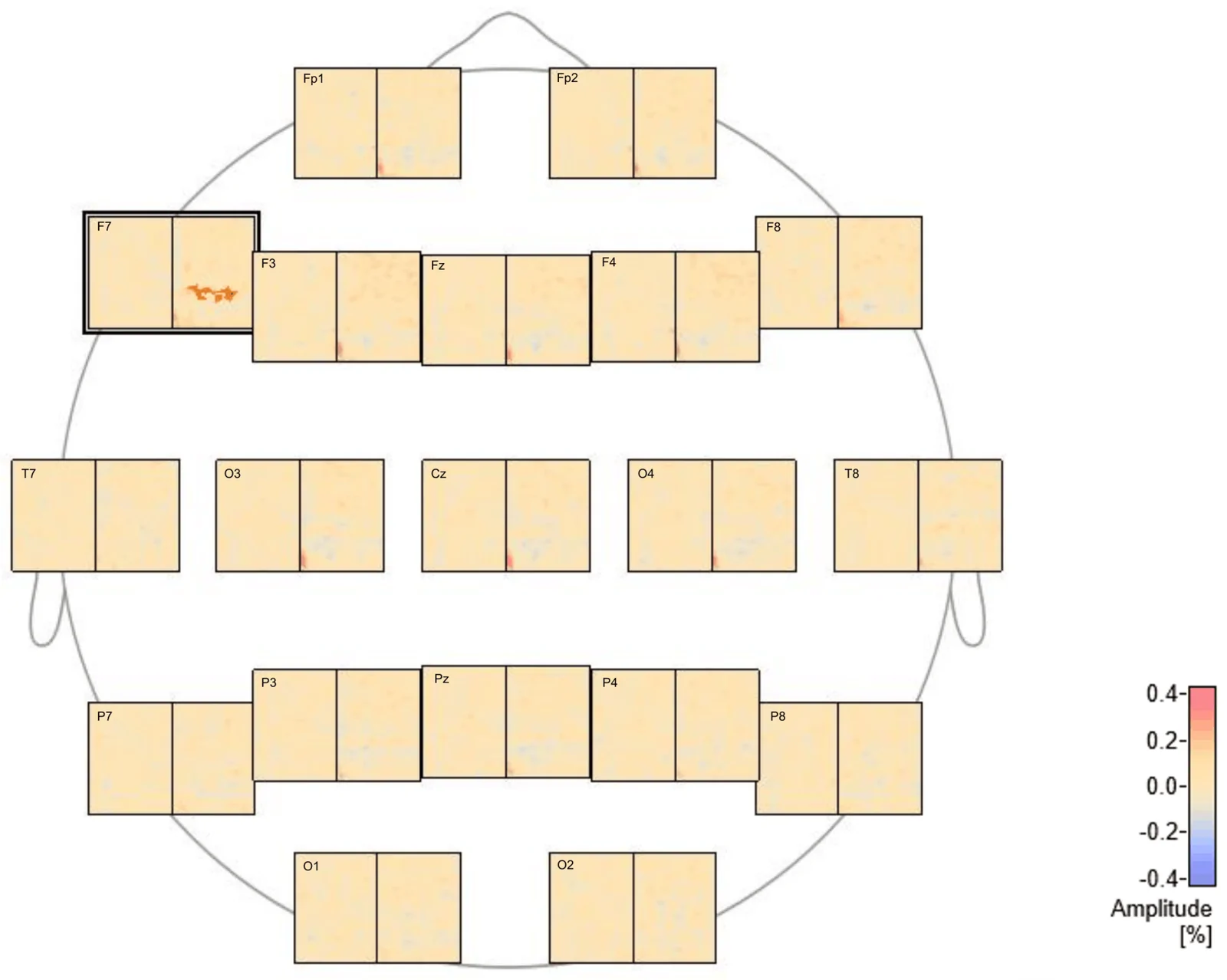
Sensor-level time–frequency results of the permutation-based correlation analysis between visual KVIQ scores and task-offset-locked VMI activity. Time–frequency maps for all 19 electrodes are shown, with each panel representing one electrode. A positive (red) correlation cluster was observed at electrode F7.

**Figure 6:**
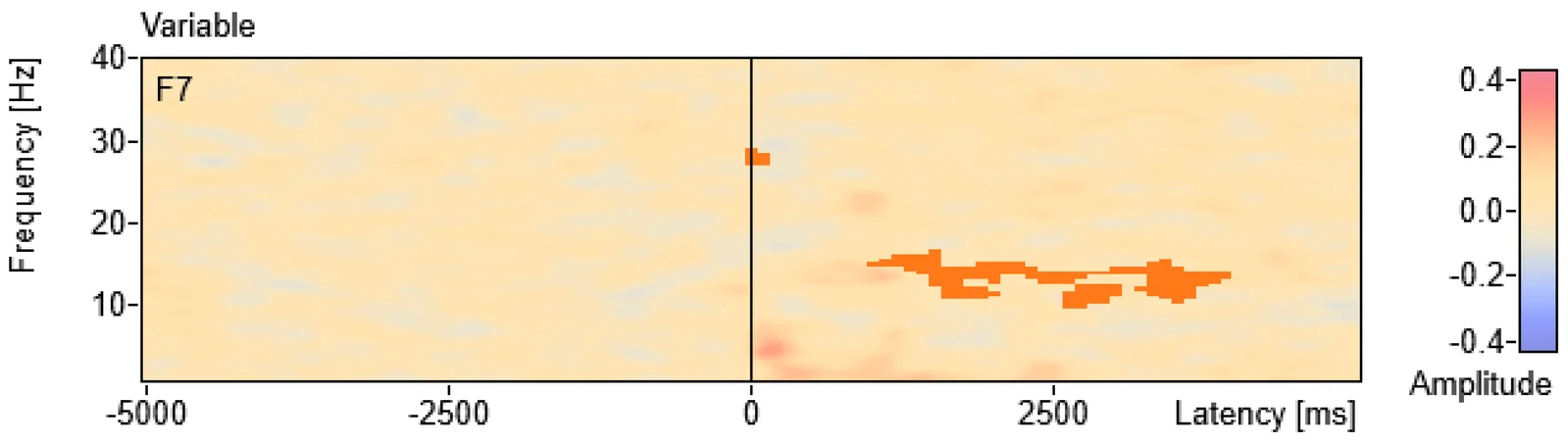
Enlarged time–frequency representation of the positive EEG–KVIQ correlation cluster identified at F7 in the task-offset-locked VMI analysis. The cluster extended from 1000 to 3900 ms and from 10 to 16.5 Hz (permutation-derived cluster-level *p* = 0.023). Time is shown on the horizontal axis and frequency on the vertical axis; 0 ms indicates task offset. The red cluster indicates a positive correlation between visual KVIQ scores and baseline-normalized relative amplitude changes (%).

**Figure 7:**
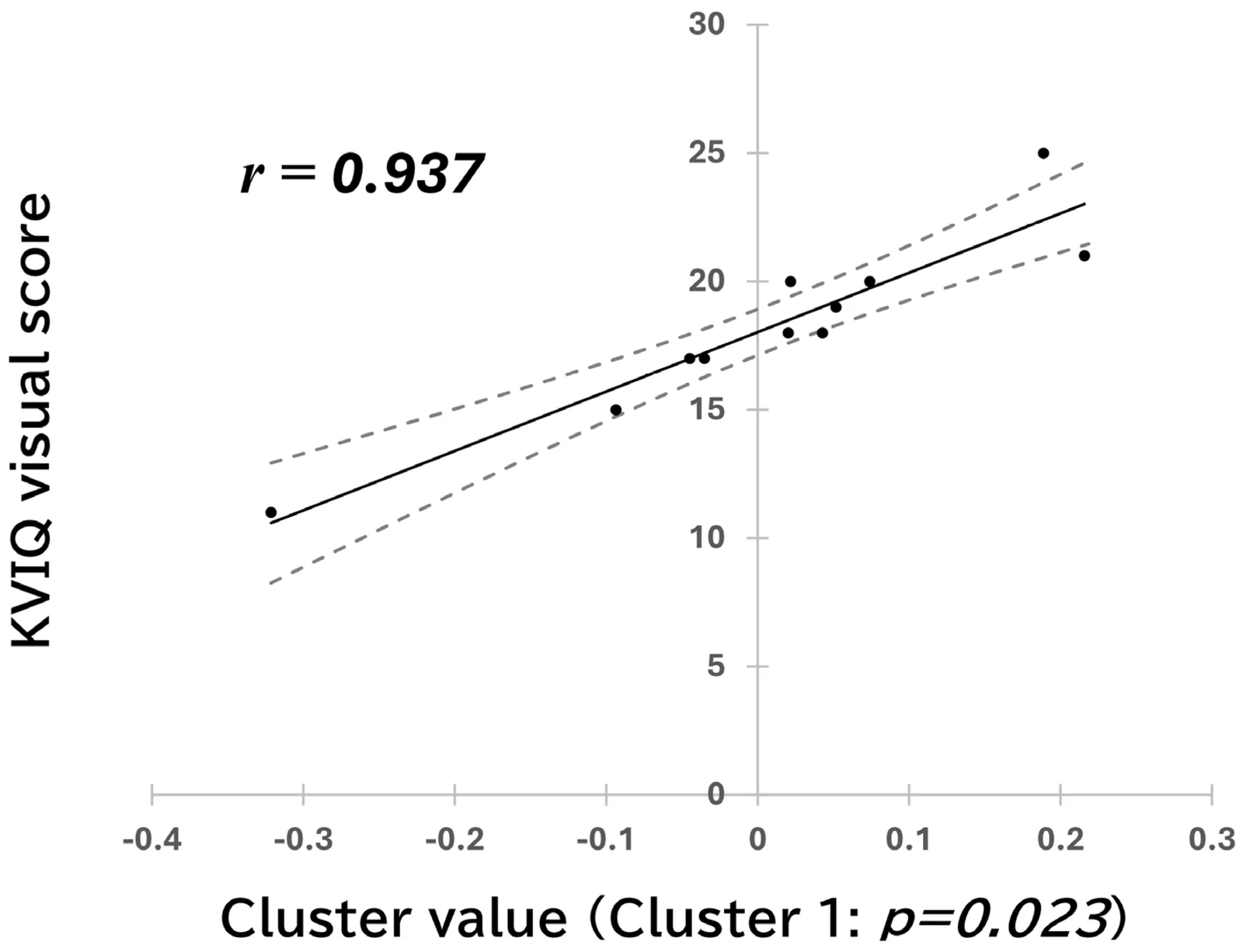
Scatter plot of visual KVIQ scores and mean baseline-normalized relative amplitude change (%) within the selected F7 time–frequency cluster in the task-offset-locked VMI analysis (1000–3900 ms; 10–16.5 Hz). The descriptive Pearson correlation was r = 0.937 (95% CI: 0.770 to 0.984); the permutation-derived cluster-level *p*-value was 0.023. The solid line indicates the least-squares regression line, and dashed lines indicate 95% confidence limits.

**Table 1:** Participant characteristics and psychometric measures.

No.	EHI Score (−100 to 100)	K Score ^1^ (5–25)	V Score ^1^ (5–25)	Total Score ^1^ (10–50)	Pre-F ^2^ (0–10)	Post-F ^2^ (0–10)	Diff. (Post-Pre)
1	100	22	19	41	5.2	6.5	1.3
2	100	17	20	37	5.2	6.7	1.5
3	100	11	18	29	3.6	5.9	2.3
4	100	14	25	39	5.9	6.8	0.9
5	100	20	18	38	6	6.5	0.5
6	100	16	15	31	7.6	9	1.4
7	100	19	21	40	7.4	7.6	0.2
8	100	21	17	38	5.7	2.2	−3.5
9	90	8	11	19	6.1	7.7	1.6
10	100	19	20	39	4.3	5.8	1.5
11	100	10	17	27	2.6	2.2	−0.4

^1^ Scores range from 5 to 25 for subscales and 10 to 50 for the total score; ^2^ Visual Analog Scale (VAS) scores range from 0 to 10. EHI, Edinburgh Handedness Inventory; K Score, Kinesthetic Score; V Score, Visual Score; Pre-F, Pre-experimental Fatigue; Post-F, Post-experimental Fatigue; Diff., Fatigue Difference. Descriptive statistics reported in the Results were recalculated directly from the participant-level values presented in this table.

## Data Availability

The data generated and analyzed during the current study are available from the corresponding author upon reasonable request, subject to ethical and privacy restrictions. The EEGLAB preprocessing scripts and BESA analysis parameters are available from the corresponding author upon reasonable request.

## Supplementary Materials

The following supporting information can be downloaded at https://www.mdpi.com/article/10.3390/brainsci16101008/s1, Figure S1: Channel-wise time–frequency representations during the ME condition. Time–frequency representations of baseline-normalized relative amplitude changes (%) during motor execution (ME) are shown for all recorded channels, arranged according to their approximate scalp locations. Each panel displays time (horizontal axis), frequency (vertical axis), and the corresponding electrode (indicated in the top-left corner). The left portion of each panel (−5000 to 0 ms) represents the pre-task rest period, whereas the right portion (0 to 5000 ms) represents the task period. These representations are provided for qualitative visualization of temporal and spectral dynamics and are not intended to represent classical ERD/ERS measures; Figure S2: Channel-wise time–frequency representations during the KMI condition. Time–frequency representations of baseline-normalized relative amplitude changes (%) during kinesthetic motor imagery (KMI) are shown for all recorded channels, arranged according to their approximate scalp locations. Each panel displays time (horizontal axis), frequency (vertical axis), and the corresponding electrode (indicated in the top-left corner). The left portion (−5000 to 0 ms) represents the pre-task rest period, and the right portion (0 to 5000 ms) represents the imagery period. These plots allow qualitative inspection of within-task temporal and spectral patterns across channels and complement the cluster-based statistical analyses reported in the main text; Figure S3: Channel-wise time–frequency representations during the VMI condition. Time–frequency representations of baseline-normalized relative amplitude changes (%) during visual motor imagery (VMI) are shown for all recorded channels, arranged according to their approximate scalp locations. Each panel displays time (horizontal axis), frequency (vertical axis), and the corresponding electrode (indicated in the top-left corner). The left portion (−5000 to 0 ms) represents the pre-task rest period, and the right portion (0 to 5000 ms) represents the imagery period. These visualizations are provided for qualitative reference only and do not imply a conventional resting-state comparison or independent statistical inference at the single-channel level.

## Abbreviations

The following abbreviations are used in this manuscript:

EEG	Electroencephalography
EHI	Edinburgh Handedness Inventory
ERD	Event-Related Desynchronization
ERS	Event-Related Synchronization
ICA	Independent Component Analysis
KMI	Kinesthetic motor imagery
KVIQ	Kinesthetic and Visual Imagery Questionnaire
ME	Motor execution
MI	Motor imagery
VAS	Visual Analog Scale
VMI	Visual motor imagery
