# Supplementary material for: Association Between EEG Activity and Motor Imagery Ability in Single-Joint Movements: A Pilot Study
Source: Brain Sci. 2026 Sep 23;16(10):1008. doi: 10.3390/brainsci16101008 (PMC13702282; doi:10.3390/brainsci16101008)
Supplement: Supplementary file 1 [file brainsci-16-01008-s001.zip › brainsci-4555220-supplementary.pdf]

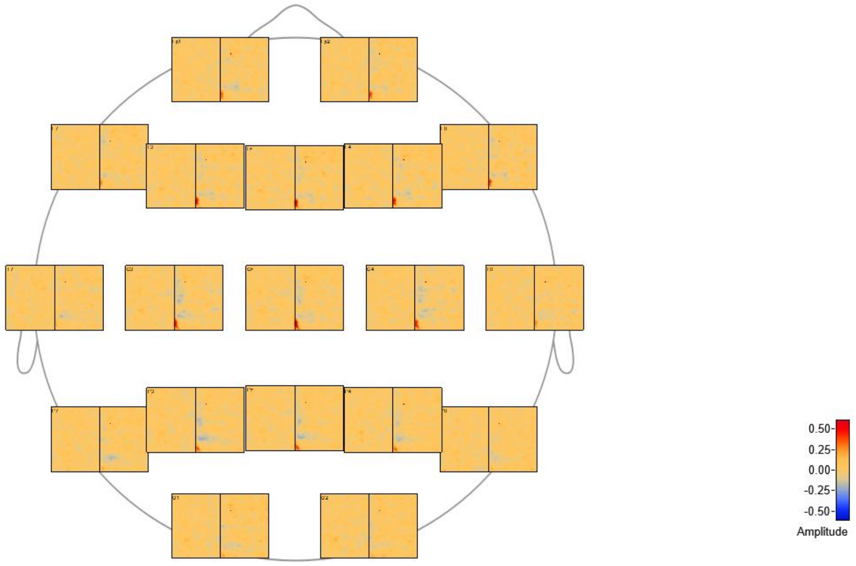

Supplementary Figure S1

Channel-wise time–frequency representations during the ME condition. Time–frequency representations of baseline-normalized relative amplitude changes (%) during motor execution (ME) are shown for all recorded channels, arranged according to their approximate scalp locations. Each panel displays time (horizontal axis), frequency (vertical axis), and the corresponding electrode (indicated in the top-left corner). The left portion of each panel (−5,000 to 0 ms) represents the pre-task rest period, whereas the right portion (0 to 5,000 ms) represents the task period. These representations are provided for qualitative visualization of temporal and spectral dynamics and are not intended to represent classical ERD/ERS measures.

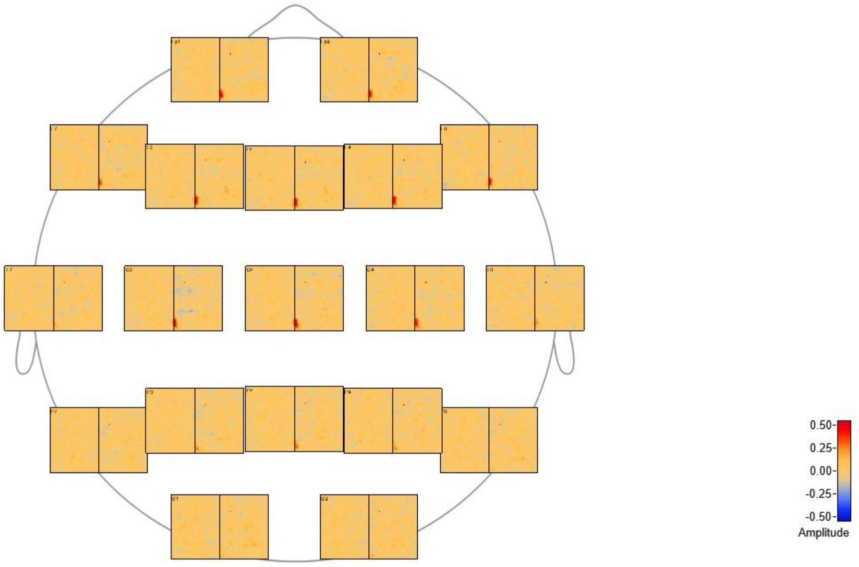

Supplementary Figure S2

Channel-wise time–frequency representations during the KMI condition. Time–frequency representations of baseline-normalized relative amplitude changes (%) during kinesthetic motor imagery (KMI) are shown for all recorded channels, arranged according to their approximate scalp locations. Each panel displays time (horizontal axis), frequency (vertical axis), and the corresponding electrode (indicated in the top-left corner). The left portion (−5,000 to 0 ms) represents the pre-task rest period, and the right portion (0 to 5,000 ms) represents the imagery period. These plots allow qualitative inspection of within-task temporal and spectral patterns across channels and complement the cluster-based statistical analyses reported in the main text.

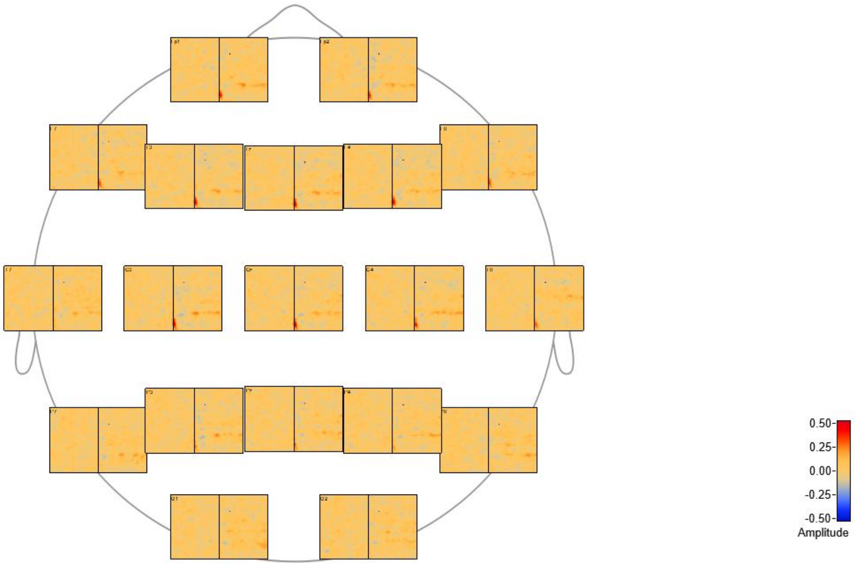

Supplementary Figure S3

Channel-wise time–frequency representations during the VMI condition. Time–frequency representations of baseline-normalized relative amplitude changes (%) during visual motor imagery (VMI) are shown for all recorded channels, arranged according to their approximate scalp locations. Each panel displays time (horizontal axis), frequency (vertical axis), and the corresponding electrode (indicated in the top-left corner). The left portion (−5,000 to 0 ms) represents the pre-task rest period, and the right portion (0 to 5,000 ms) represents the imagery period. These visualizations are provided for qualitative reference only and do not imply a conventional resting-state comparison or independent statistical inference at the single-channel level.
